# Gene-enhanced tissue engineering for dental hard tissue regeneration: (1) overview and practical considerations

**DOI:** 10.1186/1746-160X-2-12

**Published:** 2006-05-15

**Authors:** Paul C Edwards, James M Mason

**Affiliations:** 1 Creighton University School of Dentistry, Omaha, NE, USA; 2 NorthShore-Long Island JewishFeinstein Institute for Medical Research, Manhasset, NY, USA

## Abstract

Gene-based therapies for tissue regeneration involve delivering a specific gene to a target tissue with the goal of changing the phenotype or protein expression profile of the recipient cell; the ultimate goal being to form specific tissues required for regeneration. One of the principal advantages of this approach is that it provides for a sustained delivery of physiologic levels of the growth factor of interest.

This manuscript will review the principals of gene-enhanced tissue engineering and the techniques of introducing DNA into cells. Part 2 will review recent advances in gene-based therapies for dental hard tissue regeneration, specifically as it pertains to dentin regeneration/pulp capping and periodontal regeneration.

## i. Introduction

Current approaches to tissue regeneration include: (i) the use of passive three-dimensional scaffolds to provide a local environment that is conducive to new tissue formation, (ii) inductive strategies in which additional growth factors are incorporated into a scaffold/matrix to modify cell behavior, and (iii) strategies to form a vital construct of cells, either fully differentiated autologous cells of the desired type, or stem cells that have been isolated and expanded *in vitro *to restore tissue function.

Gene-enhanced tissue engineering (GETE) combines techniques of tissue engineering with gene therapy. Specifically, gene-based therapies involve delivering a specific gene to the target tissue with the goal of changing the phenotype or protein expression profile of the recipient cell [1]. This can stimulate the gene-enhanced cell and/or neighboring uncommitted cells to differentiate into the desired cell and tissue types. One of the principal advantages of this approach is that it provides for a sustained delivery of physiologic levels of the growth factor of interest. This is in contrast to protein delivery systems, which are often hampered by the short half life of the delivered protein.

The central premise underpinning this approach is the existence of a population of progenitor cells that are capable of regenerating different tissues with guidance from local cues in the wound environment. Mammalian cells are, of necessity, fully capable of forming the varied tissues and organs during initial development and growth of the organism. This regenerative ability is decreased with aging, in part the result of a decrease in production of the specific protein factors involved in regulating development of these tissues [2]. The goal of gene-enhanced tissue engineering is to reclaim this lost regenerative capacity by local delivery of cells that have been genetically-enhanced to deliver physiologic levels of these growth factors.

Based on previous studies on the regeneration of articular cartilage [3], meniscal cartilage [4], bone [5], and dermal wounds [6], it is apparent that the ideal graft material provides a source of cells capable of forming the desired tissue when suitably induced, provides a biodegradable scaffold for new tissue formation (a tissue conductive environment), and provides the appropriate signals to induce tissue formation (an inductive environment).

### a. Cellular component

One of the current tenets is that embryonic, fetal, or postnatal stem cells, isolated through complex protocols, are required for successful tissue regeneration. However, as discussed later, recent findings bring this view into question.

Stem cells are undifferentiated progenitor cells that have the capacity to self-renew, without senescence. They have the desirable property of being capable of differentiating into multiple, more specialized, cells that arise from any of the three germ layers. Stem cells can be identified based on their expression of early mesenchymal stem cell markers, such as STRO-1 and CD146/MUC18. In contrast, "progenitor" or "precursor" cells are similar to stem cells, but lack the ability to self-renew [7].

The challenge to using stem cells for tissue regeneration is to direct their differentiation along the desired pathway. Besides their ability to differentiate into multiple cell types, stem cells also interact with stromal tissue in the site of implantation, thereby repopulating the desired cell types. This property, known as homing, is believed to be regulated by signaling molecules in the local environment [8]. Additional approaches used to drive differentiation of stem cells towards the desired cell type include co-culturing with an isolate from the desired tissue type, the use of inductive cell culture conditions (e.g. supplementation of media with growth factors), and genetic modification [9,10].

#### (i) Fetal stem cells

Since the initial isolation and characterization of human embryonic stem cells from fetal blastocytes [11], there has been significant interest in the potential use of these cells for tissue regeneration. Although fetal cells appear to have great potential in the field of tissue engineering, ethical, religious and political concerns, as well as a paucity of cell lines, have dampened this enthusiasm. This has been compounded by concerns over the possibility of tissue rejection and recent evidence pointing to the potential for teratoma and teratocarcinoma formation [12]. In order to circumvent some of these issues, other potential sources of stem cells are actively being explored; including placental-derived stem cells [13] and umbilical cord blood [14].

#### (ii) Postnatal adult stem cells

Postnatal adult stem cells are multipotential cells that, under the appropriate stimulus, can be induced to develop into a number of different cell types within a specific tissue, including osteoblasts, odontoblasts, adipocytes, and neuronal-like cells [15]. Potential sources of postnatal stem cells include bone marrow-derived mesenchymal stem cells [16], muscle-derived stem cells [17] and adipose tissue-derived stem cells [18]. *In vitro*, multipotent adult stem cells appear to be capable of undergoing trans-differentiation to form other tissue types, although the significance of this phenomenon *in vivo *is unclear [7].

Overall, adult stem cells appear to be more limited in potential compared to embryonic stem cells, are difficult to harvest in sufficient quantity, and require specialized isolation techniques. As with fetal stem cells, recent evidence that raises the specter of tumorigenicity [19,20]means that additional research will be required prior to the widespread use of adult stem cells for tissue regeneration.

#### (iii) Genetically-enhanced adult cells

Recent findings bring into question the dogma that either fetal or adult stem cells are necessary for successful tissue regeneration. Certain mammalian cell types can be induced to dedifferentiate to progenitor cells when stimulated with appropriate signals [21]. A population of fibroblast-like cells isolated from fat, dermis, and gingiva have significant hard tissue regenerative potential, but only when supplemented with specific growth factors; either by genetic enhancement or through supplementation with recombinant proteins [22-27].

Our results [23,5] suggest that relatively crude preparations of fibroblast-like cells can potentially serve as a source of "differentially plastic" cells for tissue regeneration when enhanced with signaling molecules and with guidance from local cues in the wound environment. No extensive purification techniques or *ex vivo *expansion of cells are required. These are not true "stem cells" because they are only capable of regenerating tissue when genetically-enhanced with specific growth factors or morphogens.

### b. Scaffold/carrier

A biodegradable matrix is required for ease of delivery of cells to the wound site as well as to provide a three-dimensional scaffold to preserve the space of the defect in anticipation of the formation of new tissue. The ideal scaffold for tissue engineering should be relatively easy to handle, allow for the incorporation of cells, allow for the free diffusion of cells and growth factors, permit the establishment of a vascular bed to ensure survival of the implanted cells, induce a minimal inflammatory response and be ultimately biodegraded. In contrast to orthopedic situations, in which the scaffold must be strong enough to immediately tolerate considerable biomechanical forces, strength may be less of a concern when regenerating relatively small periodontal or dental defects. This however does not obviate the need to consider the effect of biomechanical effect of occlusal forces on both the dentition and surrounding tissues. From the perspective of a pulp capping agent, as well as for periodontal regeneration, it would be advantageous to have a malleable or injectable formulation, allowing it to adapt to the shape of the defect. From a regulatory standpoint, there are obvious benefits to using materials that are already approved for clinical use.

A large number of matrix materials have been employed in tissue engineering [28,29], ranging from long-lasting porous hydroxyapatite ceramics to naturally-occurring molecules of intermediate duration (e.g. alginate, collagen, chitosan), to relatively short-lasting polymers such as polyglycolic acid (PGA) and polylactic acid (PLA) and their copolymer poly(lactic-co-glycolide) (PLGA). Although this latter group of man-made polymers has been widely used, they have some inherent disadvantages. Their breakdown products can have an adverse effect on wound healing [28]. Moreover, added cells require "seeding" onto the matrix material, which requires *ex vivo *cell culturing for extended periods of time. In addition, cells often have difficulty in adhering to the polymer.

Type I collagen, a major component of bone, dental pulp and periodontal ligament, is biocompatible and has been shown to promote regeneration of hard tissue defects in various models [28]. However, collagen-based systems, by themselves, are structurally weak. The porous nature of alginate gels, a biodegradable polysaccharide, allows for the migration of cells and regulatory proteins inside the network [30]. Chemical modification designed to couple the rate of alginate degradation to new tissue formation can significantly increase the rate of tissue regeneration [31].

Constituents of the extracellular matrix play a crucial role in mineralization, cell adhesion and differentiation [32]. Methods to improve the properties of scaffold materials by introducing biomimetic motifs (e.g. the introduction of integrin-binding peptide sequences) have tremendous potential in the field of tissue regeneration [33].

Ultimately, no one matrix material will be suitable for all situations. Mixtures of two or more materials, designed to take advantage of the ideal mechanical and/or biologic properties of the individual components, will likely turn out to hold the most promise.

### c. Morphogens/growth factors

The regulation of both tooth formation and periodontal attachment formation is mediated by a complex cascade of interactions [34] involving four principal protein families: the bone morphogenetic proteins (BMPs), fibroblast growth factors (FGFs), and the mutually inhibitory hedgehog and Wnt family of proteins.

Of these, the BMPs appear to be key regulators involved in the formation of new bone and dental hard tissues. BMPs are a family of growth factors that exert their function, in part by stimulating the differentiation of non-committed precursor cells into osteoblasts [35]. A number of recent articles have reviewed the potential for BMP delivery in human bone regeneration [36-38].

## ii. Gene delivery systems

### A. Benefits and disadvantages of available systems for introducing DNA into cells

The use of GETE for tissue regeneration requires that genes are introduced into cells by some methodology. Many methods of gene transfer exist, each with their own inherent strengths and weaknesses. This section will review the benefits as well as the disadvantages of the methods currently in use as well as some of the newer techniques being developed.

The diverse techniques for introducing nucleic acids into cells are most frequently grouped under the general categories: chemical, physical, and viral. When chemical or physical means are used, these are examples of transfection. When replication incompetent viral vectors are used, the term is transduction. The introduction of DNA by means of wild type or replication competent viral vectors is termed infection.

#### a. Chemical methods

##### (i) Calcium phosphate

Calcium phosphate co-precipitation of DNA was first described over 30 years ago [39] and continues to be used today mainly due to its low cost and reasonable transfection efficiency. DNA is combined with calcium chloride and further mixed with phosphate-buffered saline and incubated at room temperature. A DNA/calcium phosphate precipitate forms which, when added drop wise to cells in culture, is endocytosed. The majority of the internalized DNA is destroyed by cytoplasmic nucleases but a small amount avoids that fate, and is transported to the nucleus where the genes are expressed transiently for 2–3 days [40]. In a small number of cells (often in the range of one per hundred thousand), the DNA can integrate into the genome randomly and if a selectable marker is present on the DNA, stably transfected cells can be obtained as clones or populations.

Advantages of this method are the low cost and simple procedure. Disadvantages include sensitivity to impurities in DNA preparations and to minor changes in pH, salt concentration, and temperature; often resulting in poor reproducibility. This method is largely being replaced by lipid-based approaches which, although more costly, generally give consistently better transfection efficiencies.

##### (ii) DEAE-dextran

Diethylaminoethyl (DEAE)-dextran was one of the first chemical reagents used for DNA transfer into cells [41]. DEAE-dextran is a cationic polymer that associates with negatively-charged nucleic acids. Excess polymer in the DEAE-dextran/DNA complex enables the complex to associate with the negatively-charged cell membrane where it is endocytosed. Transient, but not stable, expression of transferred DNA follows. Advantages of this method are low cost, simplicity, and reproducibility. Disadvantages include lack of utility for obtaining stable transfection and the fact that it is effective only for a limited number of cell types such as macrophages. DEAE-dextran has largely been replaced by newer, more versatile technologies.

##### (iii) Lipids

Artificial liposomes were first used to deliver DNA to cells as early as 1980, however, the popularity of this method increased dramatically with the advent of cationic lipids for transfection or "lipofection" as this method is now called [42]. Many types of lipids are used to lipofect cells, hence the many lipofection products on the market. In general, cationic lipids contain a positively-charged head group, such as an amine, attached to a linker group with at least two hydrophobic tail groups. The cationic portion of the lipid molecule associates with the negatively-charged nucleic acids to form a complex. The overall net positive charge of the complex allows for closer association with negatively-charged cellular membranes. Following endocytosis, the complexes appear in endosomes and are destined for destruction in lysosomes. To prevent this destruction of the DNA, the lipid mixture also contains a neutral lipid (i.e. L-dioleoylphosphatidylethanolamine; DOPE) that facilitates release of the DNA from the endosomes, thereby freeing up the DNA to make its way to the nucleus.

Advantages of this method are the ability to transfect a large number of cell types, high efficiency transient transfection, ease of use, and good reproducibility. Disadvantages include the high cost relative to calcium phosphate and DEAE-dextran, the observation that some primary cells (neurons, dendritic cells, endothelial cells) are not effectively lipofected, and the fact that lipofection is not effective for direct *in vivo *application where serum is present. Overall, lipofection still remains the most popular method for *in vitro *transfection.

##### (iv) Polymers

Organic polymers have also been used for transfection. One polycation, polyethylenimine (PEI), is an organic macromolecule with a high cationic charge density. When PEI is complexed with DNA, the DNA is condensed into positively-charged particles that interact with the negatively-charged proteoglycans on the cell surface and enter cells via endocytosis. Once inside the endosomes, the PEI causes endosomal swelling and rupture, thus releasing the DNA into the cytoplasm and ultimately into the nucleus [43].

Advantages include ease of use and high transfection efficiency in some cell types as well as applicability for *in vivo *use [44]. Disadvantages include cost and the fact that not as many cell types are transfectable with this approach as compared to lipofection.

##### (v) Proteins

Proteins and peptides are sometimes used to enhance DNA transfer by cationic lipids. Proteins such as integrins [45] and transferrin [46] and peptides such as protamine sulfate [47] and nuclear localization signal peptides [48] have been used to improve transfection efficiency in certain cell types. The disadvantage of using proteins and peptides is that they increase the cost and complexity of the system.

#### b. Physical methods

Physical and mechanical methods of gene transfer have the advantage of simplicity and avoidance of the use of chemicals or viral proteins, which can potentially elicit an immune response. Disadvantage of these approaches is that they are mainly limited to transient expression in most tissue except muscle, where longer-term expression can be obtained.

##### (i) Biolistic

Using the biolistic method, DNA is coated onto metal microparticles and "shot" into cells at high velocity by electrostatic force or gas pressure [49]. Hence, this method is sometimes referred to as a "gene gun".

Advantages of this system are the simplicity of the method, no limitations to cell type transfected, and no limit on size and number of genes transfected. The major disadvantage is low efficiency of transfection. Relatively few cells take up and express the DNA. In addition, there is potential damage to tissues when used *in vivo*. This method is currently best suited to DNA immunization applications.

##### (ii) Electroporation

Electroporation involves treatment of cells with a very rapid pulse of high voltage current that results in a perturbation of the cell membrane and transient pore formation. The DNA passes through the pores and into the cytoplasm. Advantages of this system are that it can theoretically work on any cell type and there are no size limitations to the DNA. Disadvantages include: significant effort is required to optimize the technique (duration and strength of pulse) for each cell type, a relatively high degree of cell death is observed, and transfection efficiency is often low in comparison to other methods. This method is most applicable to *in vitro *studies. However, there have been reports of *in vivo *use [50].

A variation on electroporation known as nuclear electroporation (or nucleofection) involves direct electroporation of nucleic acids into the nucleus [51]. Advantages of this approach include the ability to bypass blocks in translocation of nucleic acid from the cytoplasm to the nucleus. The system is a good alternative to viral approaches for difficult to transfect primary cell lines. Disadvantages include the high cost of the equipment and reagent buffers and the fact that optimal conditions must be identified for each cell type used. Overall, it is a good option for *in vitro *transfection when other non-viral methods fail.

##### (iii) Microinjection

Direct injection of DNA into the nucleus of a cell using glass micropipettes has mainly been used in embryonic stem cells and other cultured cell lines [52]. Advantages of this system include high transfection efficiency and the lack of size limitations on the DNA injected. Disadvantages are many. This technique requires an investment in specialized equipment and personnel training and automation of the process is costly. Microinjection is currently not practical for the majority of gene transfer applications that require the production of large numbers of transfected cells.

##### (iv) Naked DNA

Some tissues, particularly muscle, can be transfected with DNA alone without any other transfection reagents. This accidental discovery was reported in 1990 [53], when it was observed that DNA without transfection reagent actually transfected mouse muscle better than liposome/DNA complexes. This technique can be used to very effectively transfect muscle cells *in vitro*. However, it does not work well for other cell types nor does it work as well *in vivo*; possibly due to degradation of the DNA prior to cellular uptake.

#### c. Viral vectors

Viruses have evolved over millennia to efficiently introduce genes into living cells. With the technological breakthroughs in molecular biology since the 1970s, it has become possible for molecular virologists to engineer different types of infectious viruses into gene delivery systems called viral vectors. To create viral vectors, key genes in a wild type viral genome are deleted, creating a defective viral genome that is incapable of producing infectious viruses in cells (replication incompetent). Removal of the key viral genes not only renders the viral genome replication defective, but also creates the space needed for inclusion of a therapeutic or marker gene. Hence, a "viral vector" genome is created that contains viral regulatory elements, packaging signals, other viral coding sequences, as well as therapeutic genes. However, the viral vector cannot reproduce itself.

Viral vector genomes must be propagated in special cell lines called packaging cells or vector producer cell lines. In these special cell lines, the key viral genes that were removed from the viral genome are expressed separately (*in trans*) from the viral vector genome such that all of the viral proteins needed for assembly of viral particles are expressed. Thus, the viral vector producer cell line produces "viral vector particles" that contain the viral vector genome. The viral vector particles are used to deliver the therapeutic genes to target cells in a process called transduction. Once inside target cells, the vector genome expresses the therapeutic genes and any other viral genes included in the vector genome. Because some key viral genes are missing from the vector genome, the target cells cannot produce wild type viruses or more viral vector particles. Thus, viral vectors are replication incompetent.

Because so many viruses are being studied by a multitude of virologists, it can be expected that more viral-based gene delivery systems will be developed in the coming years. Although there are many viral vector delivery systems, none are ideal for all gene transfer applications. Each viral system has unique strengths and weaknesses.

##### (i) Adeno-associated virus (AAV)

AAV is a parvovirus and its genome consists of a single-stranded DNA approximately 4.7 kb in length. There are several serotypes of AAV, with AAV2 being most commonly used in gene transfer experiments.

Advantages of AAV are that they can transduce many different cell types both *in vitro *and *in vivo*, they transduce non-dividing cells as well as dividing cells, they remain episomal and, in some cell types, can express transgenes for months without integration. They have been used safely in several gene therapy clinical trials. Wild type AAV can integrate preferentially into human chromosome 19. However, viral vectors based on AAV do not integrate into the genome frequently nor do they preferentially target chromosome 19. The vast majority of people are seropositive for AAV, which is a non-pathogenic virus often associated with adenoviral infections. Different serotypes of AAV have differing cell type preferences and elicit different immune responses [54]. Disadvantages of AAV include its small packaging capacity and difficulty in producing helper-free stocks of AAV at high titer. AAV will continue to grow in popularity due to the fact that it does not integrate into the genome; a key problem with other viral systems. However large scale production remains problematic.

##### (ii) Adenovirus (Ad)

Adenoviruses (Ad) are group I double-stranded DNA viruses with linear genomes approximately 36 kbp in length. They are commonly used in gene therapy experiments.

Ad vectors have a number of advantages including the fact that they can be easily produced at high titer, they transduce a wide range of cells, they can transduce both dividing and non-dividing cells (although like other viruses, they are more effective at transducing dividing cells), they remain episomal in the nucleus and do not integrate, and they can transiently express transgenes at a high level extending from approximately 2–10 days post-transduction, after which time expression can continue at very low levels. Hence, Ad vectors are mainly useful for transient expression (7–8 days). Disadvantages of Ad vectors include the transient nature of expression if longer expression is required, and immunogenicity can be severe. It is often not possible to give repeat administrations of Ad vectors because of neutralizing antibodies elicited from earlier Ad administration. Packaging capacity is also not as large as with other systems. However, in light of the increased regulatory scrutiny given to integrating vector systems, use of Ad vector technology will likely increase. In addition, newer Ad vector systems designed to reduce immunogenicity, such as the gutless system, are continually being developed [55].

##### (iii) Herpes simplex virus (HSV)

HSV is another group I double-stranded DNA virus with a linear genome of 150 kbp in length. Advantages of HSV include its large packaging capacity (~ 30 kbp), ability to transduce a wide variety of cell types (especially those in the nervous system), and the fact that both dividing and non-dividing cells can be transduced. It can be produced at high titer and does not integrate into the genome [56]. Disadvantages include the potential for significant toxicity due to expression of viral proteins.

##### (iv) Lentivirus (lenti)

Lentivirus vectors (lentivector) belong to the group VI class of RNA viruses that reverse transcribe their RNA. These belong to the same class of retrovirus as HIV-1, the causative agent of AIDS. The most popular lentivirus vector systems are based on HIV-1 [57]. However, lentivector systems based on viruses that do not normally infect humans have also been developed.

The major advantage that lentivectors have over oncoretroviral systems is the ability to transduce non-dividing cell types as well as dividing cells. Lentivectors can also express genes long term due to the fact that they integrate into the genome. They are not very immunogenic. Because they integrate into the genome, insertional mutagenesis is now a significant concern of regulatory agencies. However, this should not be problematic for basic science research. Production systems for lenti are not as efficient as compared to other viral systems. Production of modest amounts of lentivector is labor intensive. The packaging capacity of ~8 kbp often necessitates that cDNAs be used. Transgene expression levels are relatively low.

##### (v) Oncoretrovirus (retro)

Oncoretroviruses are also group VI class RNA viruses. Retroviral vectors based on Moloney murine leukemia virus [58] are the most commonly used viral vectors in human gene therapy trials. Advantages and disadvantages of the retro system are similar to those of the lentiviral system except that retros cannot transduce non-dividing cells. Lentiviral vectors have nuclear localization signals on some of their viral proteins which act to facilitate transport of the preintegration complex through the nuclear membrane. Oncoretroviral proteins lack these signals. Therefore the oncoretroviral preintegration complex cannot access the nucleus until nuclear membrane breakdown occurs during cell division. This can be problematic for *in vivo *use because most cells are not actively dividing in the body. However, this is generally not problematic when working with cells in culture (*in vitro *applications). A major advantage of retros over lentivectors is that they are much easier to produce at high titer because stable producer lines are relatively simple to generate and they produce retroviral vector particles without need of repeated transfections. It is anticipated that regulatory approval of retros-based clinical techniques will become more problematic now that documented cases of insertional mutagenesis causing cancer have appeared [59].

##### (vi) Other viral vector systems

Polio [60], sindbis [61], and SV40 [62] are among a large group of viruses being developed for gene transfer applications. Each will have specific niche applications as viral gene delivery systems.

## iii. Practical considerations and conclusions

### A. Overview of current approaches

The principal drawback with any cell-based regenerative tissue therapy is the individualized nature of this type of approach. As currently performed, these techniques require harvesting of a portion of the patients tissue (e.g. hematopoietic bone marrow), isolation of stem cells, *ex vivo *expansion, followed by transplantation back into the patient. This is both time consuming and labor intensive.

The development of universal donor stem cell lines could potentially obviate this requirement. Prior to widespread use of such an approach, the potential for tissue rejection would have to be addressed. While this might necessitate pharmacologically-mediated immune suppression, recent evidence suggests that allogeneic adult stem cells have a number of mechanisms that allow them to circumvent host immune responses [63-65]. Moreover, cell encapsulation appears to decrease the risk of an immune response [66], possibly via antigenic masking.

Alternatively, a simplified approach could be developed that eliminates the need for extensive purification of harvested tissue. This can be accomplished by using minimally-processed harvested tissue that has been genetically-enhanced with specific growth factors (i.e. gene-enhanced tissue engineering).

### B Factors to be considered when developing new regenerative techniques

A number of issues should be considered prior to developing any tissue engineering approach for pulpal and/or periodontal regeneration.

#### (a) Regulatory issues

Early contact with government regulatory agencies such as the Food and Drug Administration in the United States (FDA) is essential to eliminate or reduce areas of potential regulatory concern. As previously discussed, principal components in any tissue regeneration product will include one or more of: 1) a cellular component, 2) a protein or gene transfer agent, and 3) a synthetic matrix to contain the first two components. The key to success is selecting individual components that will have the best likelihood of obtaining regulatory approval.

##### (i) Cells

Although each situation is different, regulatory issues for cellular components become increasingly more complex when moving from autologous to allogeneic cells. In addition to the source of cells, the cell type and the need for characterization of cells is a key issue to regulatory agencies. It is important to point out that it is not essential that cellular components be "purified". This is often incorrectly stated as an FDA requirement by companies that have patented cell-type specific purification procedures. The FDA is primarily concerned with "characterization" of the cells; one of the principal goals being to address concerns that the product is safe and consistent in formulation. Purification *per se *is not required and, in fact, attempts to develop a system requiring the isolation of specific cell types may prove detrimental to the ultimate goal of marketing a clinically viable product.

##### (ii) Growth factor delivery systems

A growth factor/protein is often included as one component of a tissue regenerative product. This can be delivered in a number of ways: as exogenously produced protein, as a gene product delivered directly via plasmid DNA or viral vector, or as part of a cellular therapy where the cells are genetically-enhanced to overexpress specific genes. Direct protein delivery is the easiest approach from a regulatory standpoint. Gene-enhanced cell delivery systems involve significantly more regulatory hurdles. The choice of gene delivery system also has important regulatory considerations. Transient expression systems are preferred over systems involving prolonged expression. Integrative vector systems, in which the transgene and/or viral vector integrates into the target cell genome, are used in situations where therapeutic gene expression is desired for prolonged periods. However, it has now been documented that integration of genes/viral vectors can result in tumor formation [59]. As a result, regulatory agencies may require that all patients receiving integrative vector systems be monitored for life as part of the clinical trial. This makes the use of integrating vector systems impractical for wide-spread use. For this reason, the use of a non-integrating vector system is preferred for transgene delivery.

##### (iii) Matrix

The matrix is used to contain the cells and/or other components of the tissue regenerative product. Tissue regenerative agents that contain a cellular component in addition to other matrix materials are classified as "combination products" by the FDA, meaning that the product is part device and part biologic. From a regulatory standpoint, it is preferable to use materials that have been previously approved for clinical use. Otherwise, the required safety studies of each individual component will make the Phase I trial safety design considerably more difficult. Where possible, matrix materials should be from non-mammalian sources (e.g. alginate).

##### (iv) Animal models

Multiple animal models may have to be developed. Before considerable effort is made in the development of animal models for pre-clinical studies, the appropriate regulatory agencies should be contacted to ascertain if the animal models chosen will be acceptable. Regulatory agencies sometimes require use of specific large animal models for certain studies and this has significant impact on project cost and logistics as many research facilities cannot house large animals. It is important that these issues be discussed with regulatory agencies prior to commencement of animal studies.

#### (b) Practical considerations

Any new technology intended for widespread clinical usage should be based, in large part, on currently existing methodologies. This will reduce the time and effort required to bring the product to market. Consideration of how the technology can be applied *en masse *should also be anticipated.

##### (i) Cellular component

Allogeneic cells must be expanded in culture, characterized and tested for genetic stability and infectious agents prior to use. Their principal advantage lies in the fact that one cell line could potentially be marketed as a single tissue regenerative product to treat many different patients. Immune rejection and genetic drift/instability of cultured cells are two issues that need to be addressed. It is also apparent that human embryonic stem cell lines can acquire genetic changes in culture and have the potential of transforming into teratomas. Consequently, for tissue regenerative procedures, the use of autologous cells is preferred over allogeneic donor cells, thereby eliminating the potential for immunological rejection and disease transmission.

In order to develop a cost-effective autologous tissue regenerative product, it is crucial to avoid techniques requiring *ex vivo *cell expansion. This necessitates using a tissue that contains an abundant amount of easily harvestable donor cells that can be obtained with minimal donor site morbidity.

##### (ii) In vivo versus ex vivo approaches

*Ex vivo *culture techniques are labor intensive, time consuming, and require that cells be extensively characterized to rule out the acquisition of genetic mutations. Direct *in vivo *application of gene transfer vectors (e.g. direct injection of the gene transfer vector into the patient) likewise remains a challenge. Practical considerations include targeting the vector to the desired target cells, avoidance of significant first pass loss in the liver or lungs, resistance to destruction by human serum complement, and demonstrated non-targeting of germ cells.

Development of novel combination approach involving *in vitro *binding of vector to host cells for a short period of time followed by reintroduction of the vector-bound cells in an implantable matrix holds significant potential. The actual cellular transduction would occur *in vivo*, but because the cells are held *in vitro *for only a short period of time to allow for vector binding, no *ex vivo *cell expansion is required.

##### (iii) Gene transfer systems

The choice of gene transfer system must be tailored to the specific needs of each application. For genetic enhancement of an autologous cell product, the efficiency of gene transfer is a paramount consideration. Since non-viral gene transfer systems tend to be inefficient, it is preferable to use a viral vector delivery system; ideally one with an established record of clinical use. Due to the regulatory issues described earlier, it is not prudent at this time to use integrating viral vectors. Since transgene expression on the order of one week may be long enough to allow for the transdifferentiation of cells, adenovirus vector system are a good option for many applications. The major drawback with adenoviral systems is the potential for significant immunogenicity to the adenoviral vector proteins. This problem can be greatly reduced through the use of matrix materials that mask these epitopes.

##### (iv) Ease of translation into a clinical protocol

Ideally, the entire process of donor tissue harvest, processing of the tissue, transduction, matrix assembly, and re-implantation of vector-bound cells should take place as a single outpatient procedure in the clinical setting, without the need for specialized equipment or extensive training.

#### C. Funding sources

Obtaining funding to transition from the research phase to commercial production of cell-based therapies can be challenging. Venture capitalists have been reluctant to invest in these firms because of historically low rates of return on investment as well as concerns over patent rights [67]. While the United States Patent and Trademark Office has issued a number of patents on human-derived cells, foreign countries have been reluctant to issue similar patent protection. These factors should be considered early on, since the ultimate objective is the development of a tissue regenerative product that will succeed both in the clinical setting and in the marketplace.

## Competing interests

The author(s) declare that they have no competing interests.

## Authors' contributions

Both authors contributed equally.
